# High Green Light Substitution Reduces Tipburn Incidence in Romaine Lettuce Grown in a Plant Factory with Artificial Lighting

**DOI:** 10.3390/plants15020208

**Published:** 2026-01-09

**Authors:** Thanit Ruangsangaram, Maitree Munyanont, Duyen T. P. Nguyen, Michiko Takagaki, Na Lu

**Affiliations:** 1 Graduate School of Horticulture, Chiba University, 648 Matsudo, Matsudo 271-8510, Chiba, Japan; rsar.thanit@gmail.com (T.R.); mtgaki@faculty.chiba-u.jp (M.T.); 2 Lamtakhong Research Station, Expert Center of Innovative Agriculture, Thailand Institute of Scientific and Technological Research, Pathum Thani 12120, Thailand; maitree_m@tistr.or.th; 3 Center for Environment, Health and Field Sciences, Chiba University, 6-2-1 Kashiwanoha, Kashiwa 277-0882, Chiba, Japan; duyentuu0406@gmail.com

**Keywords:** calcium deficiency, indoor cultivation, leaf necrosis, leafy vegetable, LED spectrum, light penetration

## Abstract

Optimizing lighting in plant factory with artificial lighting (PFAL) is essential for balancing lettuce growth and quality. Rapid plant growth in PFAL often induces physiological disorders, especially tipburn, which is associated with calcium deficiency in newly emerging leaves. This study aimed to evaluate the effects of different proportions of green light substitutions on tipburn incidence and the growth of romaine lettuce cultivated in a PFAL. Plants were grown under different proportions of green light at a total light intensity of 200 µmol m^−2^ s^−1^, consisting of 40% (G40), 60% (G60), 80% (G80), and 100% (G100) green light. The results showed that increasing the proportion of green light significantly reduced tipburn incidence from 49% to 25%, while shoot fresh weight declined by 7% and 25% when green light substitution increased to 80% and 100%, respectively. Net CO_2_ assimilation of outer leaves remained similar among G40, G60, and G80 but declined by approximately 13% under 100% green light (G100). Higher proportions of green light markedly increased calcium accumulation and chlorophyll content in the inner leaves. These results suggest that higher proportions of green light may improve the inner-leaf light environment, enhance inner-leaf physiological function, and thereby reduce tipburn incidence. Substituting 60% green light achieved a good balance between growth performance and tipburn reduction. This approach offers an effective method to mitigate tipburn and improve lettuce quality in PFALs.

## 1. Introduction

Tipburn is a significant issue in lettuce production in plant factories with artificial lighting (PFALs). It not only reduces visual appeal of harvested leaves, compromising their marketability, but also causes postharvest losses [1]. PFALs offer highly controlled environments that allow year-round production and high yields, but they can also exacerbate calcium-related disorders, such as tipburn, due to conditions that promote rapid plant growth [2,3].

Plants require calcium (Ca) for proper growth and development, as it serves as an essential structural component of cell walls and membranes and acts as a key secondary messenger in cellular signaling pathways [4]. However, unlike other nutrients, Ca is considered immobile in the phloem and is transported almost exclusively through the xylem via the transpiration stream [5]. This dependence on transpiration-driven transport leads to highly uneven Ca distribution within the plant, particularly in tissues with low transpiration rates, such as young, rapidly growing leaves. Insufficient calcium supply to these leaves leads to localized deficiencies, which can ultimately lead to tipburn, a physiological disorder characterized by necrosis along the leaf margins [6,7].

Numerous strategies have been proposed to mitigate tipburn in PFAL systems, including growth retardation, foliar Ca application, enhancing airflow to promote transpiration, and optimizing humidity [1,8,9,10]. However, these strategies are often energy-intensive and technically challenging. Consequently, optimizing light quality has emerged as an attractive alternative approach. Adjustments in light spectrum have been shown to influence plant morphology and photosynthetic efficiency, as well as regulate stomatal behavior and transpiration [11]. This, in turn, can indirectly affect calcium transport and allocation [12].

Among the various spectral regions, green light has received increasing attention due to its unique properties. While red and blue photons are absorbed efficiently by chlorophyll in the upper leaf layers, green photons penetrate more deeply into the mesophyll and inner leaf layers, where light availability is limited [13]. Although green light has a relatively low quantum yield and may reduce overall transpiration when it replaces red and blue light, evidence suggests that green light contributes to photosynthesis in inner leaves by exciting chlorophyll and accessory pigments, thereby enhancing carbon fixation [14]. By improving the inner-canopy light environment, green light may enhance local photosynthetic activity, transpiration, and metabolic processes, which could influence calcium allocation toward developing inner leaves. Additionally, appropriate levels of green light substitution can optimize canopy light distribution while maintaining overall photosynthetic productivity [15]. However, these prior studies focused primarily on the effects of green light substitution on plant growth, morphology, and photosynthetic performance, whereas the potential role of green light in alleviating tipburn via alterations in inner-leaf physiology and calcium allocation remains unexplored.

Therefore, we hypothesized that higher green light substitution would enhance calcium allocation in inner leaves and reduce tipburn incidence in romaine lettuce, and that an optimal intermediate green light fraction would minimize tipburn without compromising biomass. This study extends previous research and is innovative in emphasizing calcium distribution as a mechanism underlying green light–mediated tipburn mitigation and provides practical insights into light-based methods for improving crop quality in controlled environment agriculture.

## 2. Results

### 2.1. Tipburn Incidence and Calcium Content of the Inner Leaves

**Figure 1:**
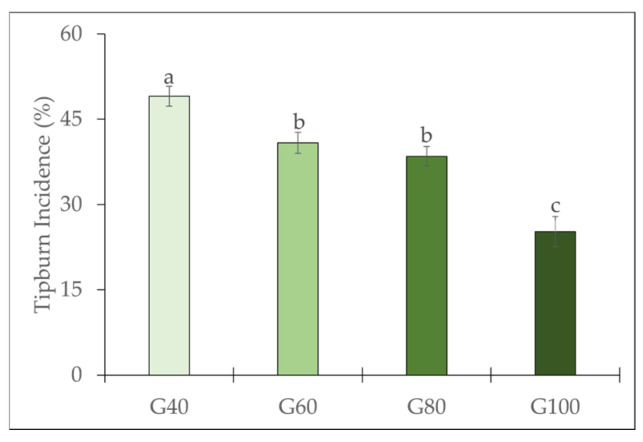
Tipburn incidence of romaine lettuce under different green light proportions. The vertical bars represent mean ± SE (*n* = 20). Different lowercase letters indicate significant differences among treatments (*p* ≤ 0.05).

**Figure 2:**
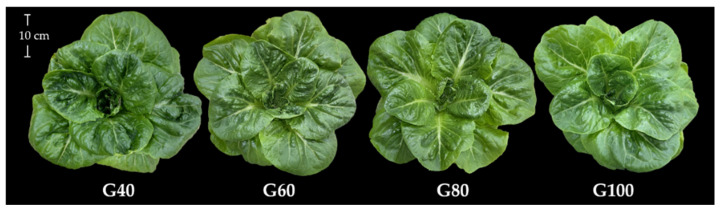
Top view of romaine lettuce grown under different green light proportions. The numbers following the letter “G” indicate the percentage of green light.

Figure 1 shows that tipburn incidence decreased significantly with increasing green light proportion, from 49% under G40 to 25% under G100. The reduction in tipburn incidence was approximately 8%, 11%, and 24% in G60, G80, and G100, respectively, compared with G40. As shown in Figure 2, visual observations revealed that romaine lettuce grown under different proportions of green light exhibited slight morphological differences. Plants grown under G40 developed larger and more expanded leaves, whereas those grown under G100 were smaller, with a looser canopy structure and slightly lighter leaf color.

**Figure 3:**
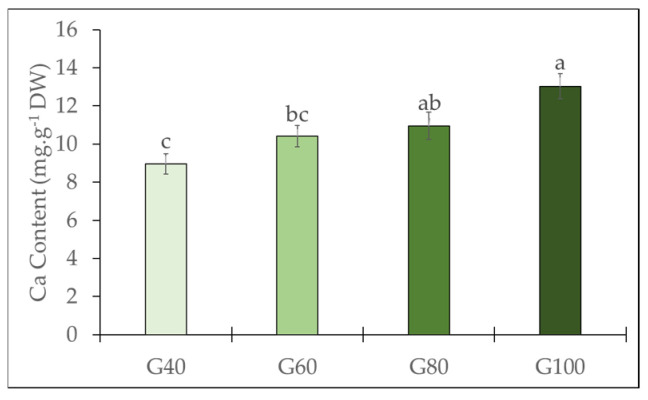
Calcium content in the inner leaves of romaine lettuce under different green light proportions. The vertical bars represent mean ± SE (*n* = 16). Different lowercase letters indicate significant differences among treatments (*p* ≤ 0.05).

Ca content in the inner leaves of romaine lettuce increased with increasing proportions of green light (Figure 3). Plants grown under the G100 treatment exhibited the highest Ca concentration (13.03 mg g^−1^ DW). This concentration was significantly higher than that observed in the G60 and G40 treatments, although it was not significantly different from the G80 treatment. The lowest Ca content was recorded under the G40 treatment (8.96 mg g^−1^ DW).

### 2.2. Plant Growth and Biomass

**Figure 4:**
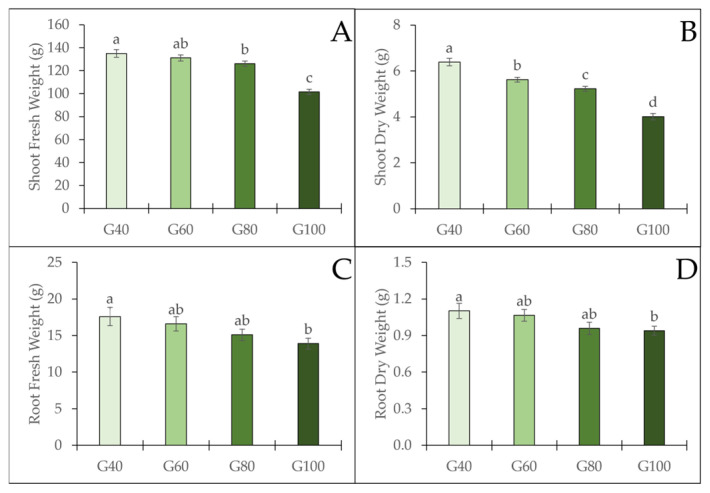
The fresh and dry weights of shoots (**A**,**B**) and roots (**C**,**D**) of romaine lettuce under different green light proportions. The vertical bars represent mean ± SE (*n* = 20). Different lowercase letters indicate significant differences among treatments (*p* ≤ 0.05).

The effects of different green light proportions on romaine lettuce growth and biomass are presented in Figure 4. Shoot fresh weight (FW) showed a decreasing trend with increasing green light proportion. Plants grown under G40 exhibited the highest shoot FW (135 g), which was not significantly different from G60 (131 g). Significantly lower values were observed in G80 (126 g) and G100 (102 g) compared with G40, showing average reductions of approximately 7% and 25%, respectively (Figure 4A). Shoot dry weight (DW) also decreased significantly, declining from 6.4 g in G40 to 4.0 g in G100 as the proportion of green light increased (Figure 4B). Additionally, the root FW and DW exhibited a similar trend to those of the shoot. Root FW declined from G40 to G80, with no significant difference among treatments. Likewise, the root DW decreased slightly from G40 to G80, with no significant difference, and reached the lowest value in the G100 treatment (Figure 4C,D). Overall, a higher proportion of green light corresponded to reduced biomass accumulation in romaine lettuce. 

**Figure 5:**
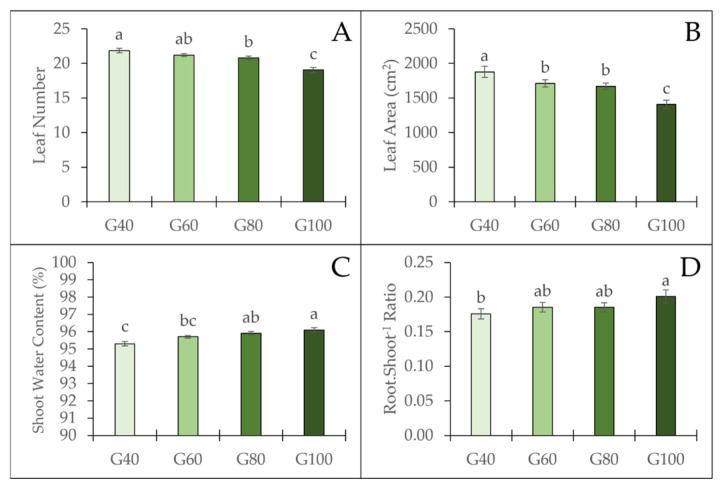
Leaf number (**A**), leaf area (**B**), shoot water content (**C**), and root-to-shoot ratio (**D**) of romaine lettuce under different green light proportions. The vertical bars represent mean ± SE (*n* = 12). Different lowercase letters indicate significant differences among treatments (*p* ≤ 0.05).

The number of leaves (Figure 5A), leaf area (Figure 5B), shoot water content (Figure 5C), and root-to-shoot ratio (Figure 5D) of romaine lettuce were significantly affected by the proportion of green light. The number of leaves decreased with increasing proportion of green light. The highest water content was observed under the G100 treatment, which was significantly greater than that of the G40 and G60 treatments, but no significant difference was found compared to the G80 treatment. The root-to-shoot ratio showed an increasing trend with increasing green light proportion, reaching the highest value under G100, which was significantly higher than G40 but not significantly different from G60 and G80. In contrast, leaf area decreased as the proportion of green light increased. Plants grown under G40 exhibited the largest leaf area, followed by G60 and G80, whereas G100 showed the smallest leaf area.

### 2.3. Gas Exchange Characteristics of Outer Leaves and Leaf Pigments

**Figure 6:**
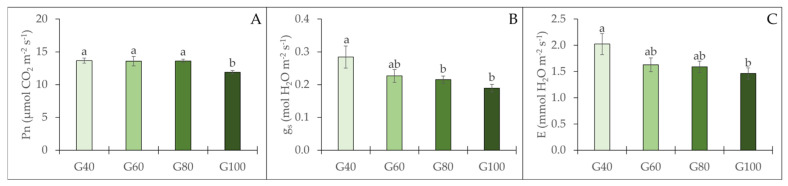
Leaf gas exchange parameters of romaine lettuce under different treatments: Pn (net CO_2_ assimilation rate, **A**), g_s_ (stomatal conductance, **B**), and E (transpiration rate, **C**). The vertical bars represent mean ± SE (*n* = 8). Different lowercase letters indicate significant differences among treatments (*p* ≤ 0.05).

The gas exchange parameters of romaine lettuce were affected by the proportion of green light (Figure 6). No significant differences were found among the G40, G60, and G80 treatments, which showed similar Pn values. However, the Pn value decreased markedly when plants were exposed to 100% green light, resulting in the lowest recorded value. Additionally, both stomatal conductance (g_s_) and transpiration rate (E) declined with increasing proportions of green light (Figure 6).

**Figure 7:**
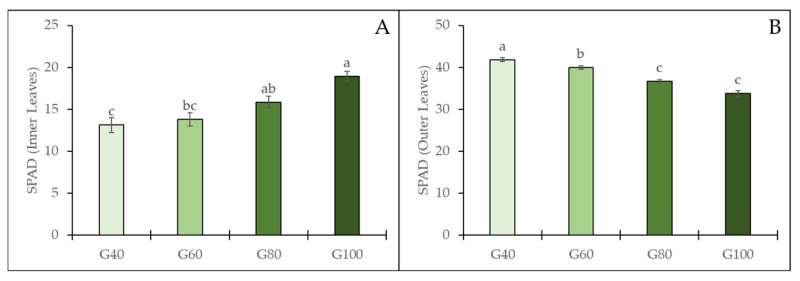
SPAD values of inner (**A**) and outer (**B**) leaves of romaine lettuce under different green light proportions. The vertical bars represent mean ± SE (*n* = 20). Different lowercase letters indicate significant differences among treatments (*p* ≤ 0.05).

The soil plant analysis development (SPAD) values varied depending on the proportion of green light (Figure 7). The SPAD value of inner leaves increased significantly by 46% from G40 to G100, indicating a greater chlorophyll accumulation within the inner canopy under higher green light levels. In contrast, the SPAD values of outer leaves decreased significantly with increasing green light proportion, showing a 19% reduction from G40 to G100.

**Figure 8:**
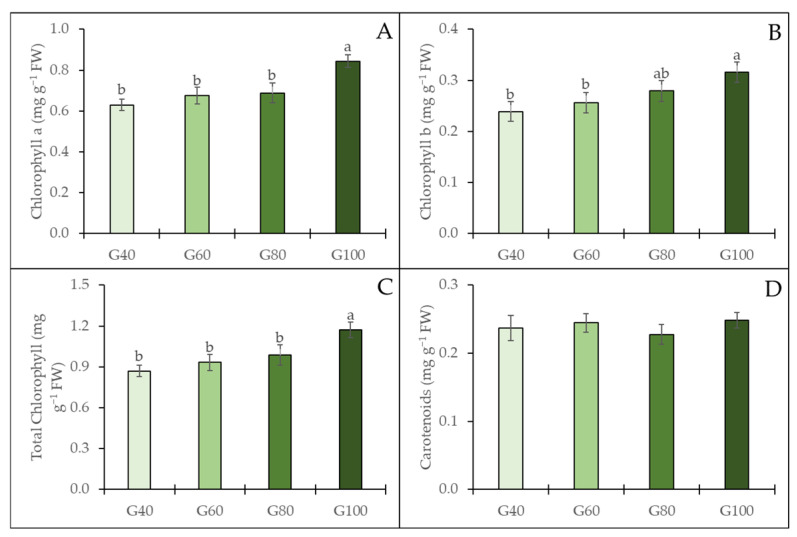
The chlorophyll a (**A**), chlorophyll b (**B**), total chlorophyll (**C**), and carotenoids (**D**) in the inner leaves of romaine lettuce grown under different green light proportions. The vertical bars represent the mean ± SE (*n* = 16). Different lowercase letters indicate significant differences among treatments (*p* ≤ 0.05).

The chlorophyll content of the inner leaves of romaine lettuce was significantly influenced by the proportion of green light treatments (Figure 8). Chlorophyll a, chlorophyll b, and total chlorophyll contents all increased with higher proportions of green light. There was no significant difference among the G40, G60, and G80 treatments with the highest values observed under the G100 treatment. However, the carotenoid content showed no significant differences among treatments. These results indicate a tendency for increasing chlorophyll accumulation in the inner leaves under high proportions of green light, whereas carotenoid levels remained relatively stable across all treatments.

## 3. Discussion

### 3.1. Effects of Green Light Substitution on Tipburn Incidence and Plant Growth

Substituting green light influenced both tipburn incidence and plant growth in romaine lettuce cultivated in a PFAL. Tipburn incidence was reduced by 8%, 11%, and 24%, while the average shoot fresh weight of plants decreased by approximately 1% (4 g), 7% (9 g), and 25% (33 g) under G60, G80, and G100, respectively, compared with G40. Reducing tipburn without compromising yield remains an important challenge in lettuce production [1,16]. In this study, increasing the proportion of green light resulted in a significant reduction in tipburn incidence; however, plant yield decreased when the proportion of green light exceeded 60%. It is well known that there is a positive correlation between plant growth rate and tipburn incidence [1,2,16]. The mitigation of tipburn incidence under green LED lighting can be attributed to the distinctive properties of green light. On the one hand, green light drives photosynthesis at a lower rate than red or blue light when acting alone, and a high fraction of green light may therefore reduce the growth rate of the plants. Moreover, because green light has higher transmittance and scattering properties than red or blue wavelengths, it can penetrate deeper into the plant canopy [17,18], potentially enhancing photosynthesis and transpiration in the inner leaf areas where tipburn is more likely to occur.

This study demonstrated that optimizing green light at a 60% substitution rate under a total PPFD of 200 µmol m^−2^ s^−1^ significantly decreased tipburn incidence while maintaining comparable yields. However, increasing total light intensity is known to accelerate plant growth, and rapid growth is associated with a higher risk of tipburn. Although a 60% green light replacement was optimal under the light intensity tested in this study, higher light intensities (e.g., ≥200 µmol m^−2^ s^−1^) are sometimes applied in PFALs and may modify green light penetration within the canopy and its physiological effects. Consequently, the optimal proportion of green light may differ from 60% under higher total light intensities. A limitation of the present study is that only a single PPFD level was examined, and the effects of green light replacement were not evaluated across a wider range of light intensities. In addition, the responses observed here may be species- or cultivar-specific. Therefore, further studies are needed to determine the optimal green light ratio across different light intensities and crop species.

Moreover, the increased shoot water content, root-to-shoot ratio, and decreased total leaf number observed at high green-light proportions may be a consequence of slower shoot growth, accompanied by a relative shift in biomass allocation toward roots, potentially reflecting an adaptive response to suboptimal light quality.

### 3.2. Effects of Green Light Substitution on Gas Exchange Characteristics and SPAD Values of Outer Leaves

Green light substitution had a significant effect on the gas exchange parameters of romaine lettuce. The net CO_2_ assimilation rate (Pn) was lowest under the G100 treatment, whereas no significant differences were observed among the G40, G60, and G80 treatments. It is reported that the reduction in Pn under pure green light corresponds to its lower quantum yield when acting alone without red and blue wavelengths [17], which may consequently lead to reduced growth and biomass accumulation. Meanwhile, the declining stomatal conductance (g_s_) and transpiration rate (E) observed in this study with increasing proportions of green light were negatively correlated with relative shoot water content, suggesting a conservative water use strategy. Green light’s dual signaling mechanism explains this pattern: at the molecular level, abscisic acid (ABA) mediates stomatal closure by activating anion channels (*SLAC1*/*ALMT12*) and promoting K^+^ efflux via *GORK*, depolarizing guard cell plasma membranes and reducing gs and E by 50–90% within minutes; furthermore, green light has been shown to upregulate ABA biosynthesis (*NCED1*/*ABA2* expression increased, *CYP707A* expression decreased) via HY5 downregulation, even under well-watered hydroponics [19]; at the hydraulic level, reduced E relative to uptake floods intercellular air spaces, diluting guard cell wall K^+^, and blocking mesophyll vapor-phase opening signals [20]. Consistent with our findings, Park et al. [21] reported that green light (520 nm) produced the highest shoot moisture content (96.7%) in C. ‘Highway Ruby’ coleus compared to red or other spectra, while a meta-analysis (136 datasets across 17 crops) confirms that green light reduces g_s_ by 15% and increases intrinsic water use efficiency (iWUE) by 15%, resulting in 4% higher fresh weight due to water retention [22]. Trials on cucumber and melon with LEDs show 15–40% increases in ABA and GABA levels with 25–50% green light, preserving relative water content (RWC) 5–15% higher via smaller stomatal apertures without a photosynthetic penalty [23,24]. We also observed that stomatal densities were higher under higher proportions of green light (Figures S1 and S2). According to Li et al. [14], stomatal density increased under higher green light substitution, whereas stomatal aperture decreased. In our study, the SPAD values of the outer leaves decreased under high green light proportion conditions, indicating a reduction in chlorophyll content. Because chlorophyll content is closely related to photosynthetic capacity [25], increasing the proportions of green light (reducing the amounts of red and blue lights) likely limited chlorophyll synthesis in outer leaves, resulting in lower net photosynthetic rates and slower plant growth.

### 3.3. Effects of Green Light Substitution on Calcium Content and Leaf Pigments in Inner Leaves

The calcium content of the inner leaves increased with higher proportions of green-light substitution, demonstrating that green light enhances calcium accumulation within the inner canopy and contributes to reduced tipburn incidence. Given that calcium is largely immobile and relies entirely on transpiration-driven xylem transport [5], the deeper penetration of green light may influence inner-leaf physiological functions associated with enhanced calcium accumulation; however, direct evidence supporting changes in inner-leaf transpiration and stomatal behavior has not been verified in the present study. Accordingly, the observed increases in calcium concentration, chlorophyll content, and SPAD values of inner leaves suggest enhanced local photosynthetic activity, potentially involving transpiration and metabolic processes, which may influence calcium allocation toward developing inner leaves. As a result, adequate calcium reinforces expanding leaf tissues and prevents marginal cell collapse by reducing ion and metabolite leakage, a physiological disturbance characteristic of tipburn [26,27]. Beyond its structural role, calcium also contributes to the regulation of chloroplast stability by supporting the integrity of pigments and chlorophyll through calcium-mediated signaling [28,29]. Thus, calcium and chloroplasts act cooperatively, with sufficient calcium maintaining chloroplast function, while compromised chlorophyll integrity may disrupt calcium functions. Building on this interaction, under high green light conditions, higher calcium accumulation, together with increased chlorophyll content in the inner leaves, may be linked to improved inner-leaf physiological activity and thereby reduced tipburn development.

Accordingly, the enhancement of calcium allocation to inner leaves by green light may not be attributable solely to changes in whole-plant transpiration, but instead to modifications in canopy light distribution and improvements in inner-leaf physiological function. Consequently, the reduction in tipburn observed at higher green-light proportions likely arises from the combined effects of altered canopy light distribution, inner-leaf physiological responses, and reduced calcium demand associated with slower shoot growth.

## 4. Materials and Methods

### 4.1. Plant Materials and Seedling Preparation

Romaine lettuce (*Lactuca sativa* L. var. longifolia, cos lettuce, Takii Seed Co., Ltd., Kyoto, Japan) seeds were sown in polyurethane sponge mats, and the boxes were covered with transparent lids to maintain humidity during seed germination. After 48 h, the lids were removed, and seedlings were exposed to white LEDs lighting at a photosynthetic photon flux density (PPFD) of 150 µmol m^−2^ s^−1^ under a 12 h photoperiod at 20 °C. To keep adequate moisture during germination, water was sprayed into the boxes every two days. At 14 days after sowing, uniform seedlings with two true leaves were selected and transplanted into a nutrient film technique (NFT) hydroponic system within a PFAL. The planting density was 44 plants m^−2^, with 20 seedlings arranged on each 100 × 45 cm foam panel.

### 4.2. Growth Conditions and Experimental Setup

After transplanting, seedlings were cultivated for 28 days, corresponding to 42 days after sowing (DAS), using the Enshi formula nutrient solution [30]. The electrical conductivity (EC) was maintained at 1.6 dS m^−1^ by replenishing the nutrient solution and water every three days, consistently, and the pH was kept at 6.5 throughout the entire crop cycle. Environmental conditions in PFAL were controlled at 20 °C air temperature, 60% relative humidity (RH), and 1000 µmol mol^−1^ carbon dioxide (CO_2_) concentration. All parameters were continuously monitored using data loggers to ensure stable growth conditions.

**Figure 9:**
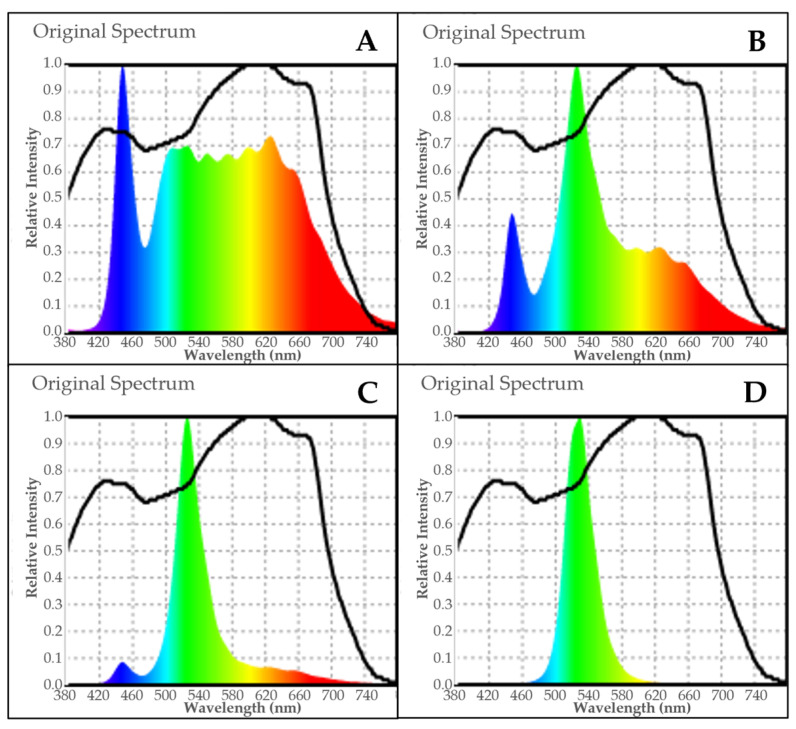
The spectral distribution of light in this experiment: G40 (**A**), G60 (**B**), G80 (**C**), and G100 (**D**). The letter G indicates the green light and the number represents the percentage of green light.

Lighting conditions were set at a PPFD of 200 µmol m^−2^ s^−1^ across all treatments, provided by white LEDs (Nova Luce series GM-BA1200M-30HR/WS, GGG Co., Ltd., Osaka, Japan) and monochromatic green LEDs (Sananbio series ZK3-TB18-SR01, peak at 526 nm, Sananbio Technology Co., Ltd., Xiamen, China), with a 16 h photoperiod per day. To achieve the designed green-light proportions and even distribution of light intensity, a light spectrum meter (Lighting Passport Pro, AsenseTek Inc., Taipei, Taiwan) was used to measure average values in each treatment (Figure 9 and Table 1) and adjust the light distribution accordingly. Natural sunlight contains approximately 35–36% green light (500–600 nm) of the total photosynthetic photon flux (400–700 nm) [31]. Based on this proportion, romaine lettuce was cultivated after transplanting in a four-tier cultivation system, in which each tier was assigned one green light proportion of 40%, 60%, 80%, and 100%, designated as G40 (control), G60, G80, and G100, respectively.

### 4.3. Measurements

#### 4.3.1. Growth Parameters

Plants were harvested at 42 DAS. The shoots and roots of romaine lettuce were separated, and the fresh weights of both parts were recorded. The roots were gently spin-dried to remove the remaining nutrient solution and blotted dry using paper towels before weighing. Subsequently, the fresh shoot and root samples were placed in paper envelopes and dried in a hot-air oven at 80 °C for five days to measure dry weights. The obtained shoot dry weight from the same plant was then used to calculate the water content (WC) according to the following equation:

$$WC\;(\%)=\frac{FW-DW}{FW}\times 100$$

where FW is the fresh weight and DW is the dry weight of the shoots.

Another set of plants was used to measure leaf area using a portable leaf area meter (LI-3000C, LI-COR Biosciences, Lincoln, NE, USA). The results were reported in square centimeters (cm^2^).

#### 4.3.2. Tipburn Evaluation and Calcium Content Analysis

At harvest, the total number of leaves (longer than 2 cm) was counted before separating those exhibiting tipburn symptoms. Tipburn incidence was evaluated based on the presence of visible symptoms, including leaf-tip chlorosis, necrotic lesions along the leaf margin, and leaf deformation. The percentage of tip burn leaves was calculated using the following equation [32,33]:

$$\mathrm{Tipburn}\;\mathrm{incidence}\;(\%)=\frac{\mathrm{Number}\;\mathrm{of}\;\mathrm{tipburn}\;\mathrm{leaves}}{\mathrm{Total}\;\mathrm{number}\;\mathrm{of}\;\mathrm{leaves}}\times 100$$

To determine the calcium content in leaves, 10 fresh inner leaves from the same plant were collected and oven-dried for analysis. The dried samples were finely ground using a mortar and pestle. A 0.2 g portion of the powdered sample was placed into a 0.5 mL crucible and ashed in a muffle furnace at 550 °C for 72 h. After ashing, each sample was dissolved in 2 mL of 2 M hydrochloric acid (HCl) within the same crucible. The resulting solution was diluted with distilled water to a final volume of 50 mL in a centrifuge tube. Next, a 1 mL aliquot of this solution was filtered through a 13 mm hydrophilic nylon membrane filter (pore size 0.45 μm) and further diluted by adding 9 mL of distilled water. The final diluted samples were used to determine calcium concentrations with an iCAP 6000 series inductively coupled plasma–optical emission spectrometer (Thermo Fisher Scientific K.K., Tokyo, Japan).

#### 4.3.3. Leaf Gas Exchange and Photosynthetic Pigments

One week before harvest (35 DAS), gas exchange parameters, including the net CO_2_ assimilation rate (Pn), stomatal conductance (g_s_), and transpiration rate (E), were measured using a portable photosynthesis system (LI-6400XT; LI-COR Inc., Lincoln, NE, USA). All measurements were performed on fully expanded leaves representative of each plant under light and environmental conditions identical to those of the cultivation system.

After harvesting, a handheld chlorophyll meter (SPAD-502 Plus, Konica Minolta, Tokyo, Japan) was used to measure SPAD values. Measurement was taken from both inner leaves (between the 3rd and 5th leaves) and outer leaves (between the 13th and 15th leaves), and the average value from three leaves was used to represent each sample. For photosynthetic pigment analysis, two fresh leaf discs (0.5 cm^2^ each) from the inner leaves (no. 3 and 4) of the same plant were punched and placed into 5 mL vials. Next, 2 mL of N, N-dimethylformamide (DMF) was added, and the samples were incubated in the dark at 4 °C for 36 h. The absorbance of the extracts was then measured at 480, 645, and 663 nm using a spectrophotometer (SH-1300 Lab, Corona Electric Co., Ltd., Hitachinaka, Ibaraki, Japan). Pigment concentrations were calculated according to the equations described below and expressed as milligrams per gram of fresh weight (mg g^−1^ FW) [34,35].

Chl b (μg mL^−1^) = 20.78A_647_ − 4.88A_664_,

Total Chl (μg mL^−1^) = 17.67A_647_ + 7.12A_664_,

Total carotenoid (μg mL^−1^) = (1000A_480_ − 1.12Chl a − 34.07Chl b)/245

where $A_{480}$, $A_{647}$, and $A_{664}$ represent the absorbance of the extract at 480, 647, and 664 nm, respectively.

### 4.4. Statistical Analysis

The experiment was conducted using a completely randomized design (CRD) and was repeated twice as independent cultivation cycles. In each cycle, ten plants per treatment were sampled to evaluate growth, SPAD values, total leaf number, and tipburn incidence, while eight plants were used to measure leaf gas exchange, photosynthetic pigments, and calcium content. Six plants were selected to determine biomass and leaf area. Data were combined from both cycles and analyzed using analysis of variance (ANOVA). Treatment means were compared using Duncan’s multiple range test in IBM SPSS Statistics (Version 23, Armonk, NY, USA). Statistical significance was set at *p* < 0.05.

## 5. Conclusions

This study demonstrated that substituting 60% green light significantly reduced tipburn incidence in romaine lettuce grown in a PFAL while maintaining yields comparable to those under 40% green light. However, increasing the proportion of green light to above 60% resulted in a decline in plant biomass and leaf area. Net CO_2_ assimilation remained similar among treatments, except under 100% green light, whereas increasing the proportion of green light trended to reduce stomatal conductance and transpiration. In addition, SPAD values decreased in the outer leaves but increased in the inner leaves when plants were grown under higher proportions of green light. The reduction in tipburn incidence corresponded with increased calcium accumulation and higher chlorophyll content in the inner leaves. Thus, under the environmental conditions of our experiment and cultivar tested, incorporating 60% green light is effective in mitigating tipburn while maintaining productivity in PFAL. The optimal proportion of green light may vary depending on the cultivar, light intensity, and photoperiod. To address the limitations of the present study, future research should evaluate a wider range of cultivars and lighting conditions.

## Figures and Tables

**Table 1:** Light intensity and proportion of green light in each treatment.

Treatments	Photosynthetic Photon Flux Density; PPFD (μmol m^−2^ s^−1^)				% Green Proportion
	Blue (400–499 nm)	Green (500–599 nm)	Red (600–700 nm)	Total (400–700 nm)	
G40 (Control)	42	81	82	205	40
G60	26	122	59	207	59
G80	17	165	24	206	80
G100	6	195	3	204	96

## Data Availability

The original contributions presented in this study are included in the article/Supplementary Material. Further inquiries can be directed to the corresponding author.

## Supplementary Materials

The following supporting information can be downloaded at https://www.mdpi.com/article/10.3390/plants15020208/s1, Figure S1: Leaf epidermal micrographs under different green light proportions; Figure S2: Stomatal density under different green light proportions.
