# Supplementary material for: High Green Light Substitution Reduces Tipburn Incidence in Romaine Lettuce Grown in a Plant Factory with Artificial Lighting
Source: Plants (Basel). 2026 Jan 9;15(2):208. doi: 10.3390/plants15020208 (PMC12845428; doi:10.3390/plants15020208)
Supplement: Supplementary file 1 [file plants-15-00208-s001.zip › plants-4040211-supplementary.pdf]

Supplementary Data

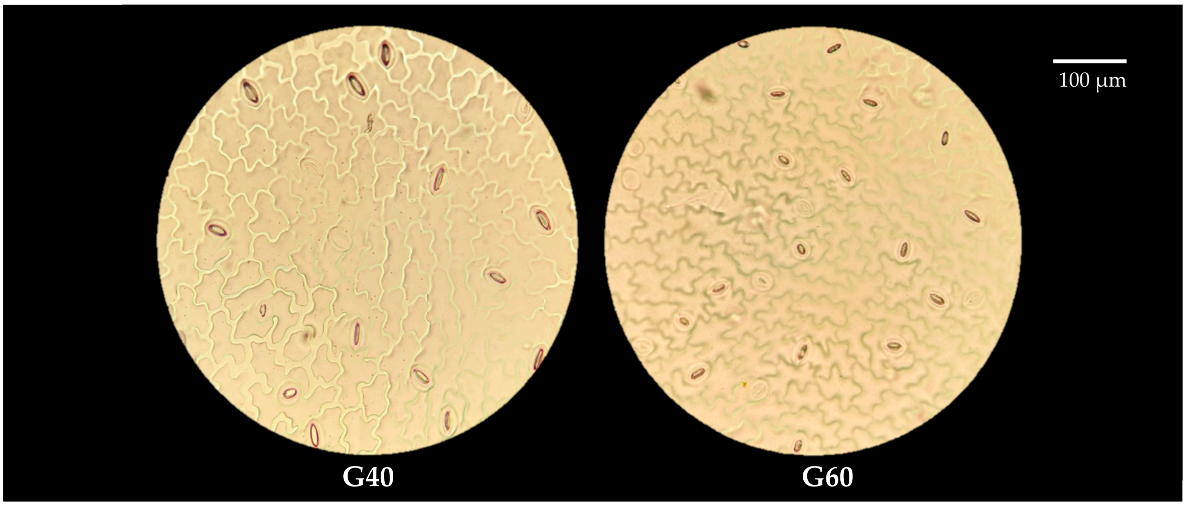

Figure S1. Leaf epidermal micrographs under G40 (left) and G60 (right). G indicates green light, and the number following G represents its percentage in the light spectrum.

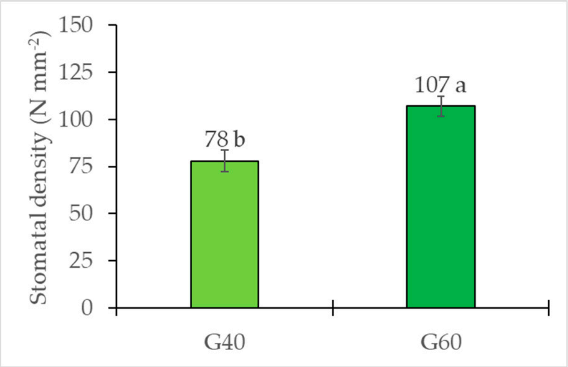

Figure S2. Stomatal density under different green proportions. G indicates green light, and the number following G represents its percentage in the light spectrum. Data are shown as mean ± SE, n = 3.
